# Supplementary material for: Predicting Remission among Perinatal Women with Depression in Rural Pakistan: A Prognostic Model for Task-Shared Interventions in Primary Care Settings
Source: J Pers Med. 2022 Jun 27;12(7):1046. doi: 10.3390/jpm12071046 (PMC9320748; doi:10.3390/jpm12071046)
Supplement: Supplementary file 1 [file jpm-12-01046-s001.zip › jpm-1740993-supplementary.pdf]

## Supplementary Table S1: Prognostic tool for predicting probability for remission of perinatal depressive symptoms

| Statement | Response |
|---|---|
| Type of intervention offered to the trial participants | 0 = Enhanced care as usual<br>1 = Thinking Healthy Programme |
| What is the structure of your family? | 0 = Nuclear<br>1 = Joint |
| Does the children's grandmother live with them? | 0 = No<br>1 = Maternal |
| Is the mother empowered (Please, choose yes if the respondent has autonomy in spending on food items and accessing healthcare?) | 0 = No<br>1 = Yes |
| On the scale given below, how would you rate the level of prosperity of this household, relative to the overall prosperity in your area? | **Richest 1 2 3 4 5 Poorest** |
| Please, rate the severity of following depressive symptoms experienced by the respondent in the last one month. | |
| **Symptom dimensions of core-emotional symptoms** | |
| Depressed mood<br><br>(Gloomy attitude, pessimism about the future, feeling of sadness, tendency to weep) | 0 = Absent<br>1 = These feeling states indicated only on questioning<br>2 = The feeling states spontaneously reported verbally<br>3 = Communicates feeling states nonverbally - i.e., through facial expression, posture, voice, and tendency to weep<br>4 = Patients reports VIRTUALLY ONLY these feeling states in his spontaneous verbal and nonverbal communication |
| Work and activities | 0 = No difficulty<br>1 = Thoughts and feelings of incapacity, fatigue or weakness related to activities, work, or hobbies<br>2 = Loss of interest in activity, hobbies or work - either directly reported by the patient, or indirect in listlessness, indecision, and vacillation (feels she has to push self to work or activities)<br>3 = Decrease in actual time spent in activities or decrease in productivity<br>4 = Stopped working because of present illness |

| | |
|---|---|
| Loss of appetite | 0 = None<br>1 = Loss of appetite but eating without encouragement. Heavy feeling in abdomen.<br>2 = Difficulty eating without urging. Requests or requires laxatives or medication for bowels or medication for G. I. symptoms |
| Somatic Anxiety (Gastrointestinal, indigestion, cardiovascular, palpitations, headaches, respiratory, genitourinary, etc.) | 0 = Absent<br>1 = Mild<br>2 = Moderate<br>3 = Severe<br>4 = Incapacitating |
| Psychiatric Anxiety | 0 = No difficulty<br>1 = Subjective tension and irritability<br>2 = Worrying about minor matters<br>3 = Apprehensive attitude apparent in face or speech<br>4 = Fears expressed without questioning |
| **Symptom dimension of somatic symptoms** | |
| Suicide | 0 = Absent<br>1 = Feels life is not worth living<br>2 = Wishes she were dead or any thoughts of possible death to self<br>3 = Suicide ideas or gesture<br>4 = Attempts at suicide (any serious attempt rates 4) |
| Loss of weight | 0 = No weight loss<br>1 = Probable weight loss associated with present illness<br>2 = Definite (according to patient) weight loss |
| Retardation (Slowness of thought, speech, and activity; apathy; stupor) | 0 = Normal speech and thought<br>1 = Slight retardation at interview<br>2 = Obvious retardation at interview<br>3 = Interview difficult<br>4 = Complete stupor |
| Somatic symptoms general (Heaviness in limbs, back, or head; diffuse backache; loss of energy and fatigability) | 0 = None<br>1 = Heaviness in limbs, back or head. Backaches, headaches, muscle aches. Loss of energy and fatigability<br>2 = Any clear-cut symptom rates 2 |
| Hypochondriasis | 0 = Not present<br>1 = Self-absorption (bodily)<br>2 = Preoccupation with health |

| | |
|---|---|
| | 3 = Frequent complaints, requests for help, etc.<br>4 = Hypochondriacal delusions |
| **Symptom dimension of insomnia** | |
| Insomnia during early night | 0 = No difficulty falling asleep<br>1 = Complains of occasional difficulty falling asleep - i.e., more than 1/2 hour<br>2 = Complains of nightly difficulty falling asleep |
| Insomnia during middle of the night | 0 = No difficulty<br>1 = Patient complains of being restless and disturbed during the night*<br>2 = Waking during the night - any getting out of bed (except for purposes of voiding) |
| Late insomnia | 0 = No difficulty<br>1 = Waking in early hours of the morning but goes back to sleep<br>2 = Unable to fall asleep again if she gets out of bed |
